# The adaptive immune response in cardiac arrest resuscitation induced ischemia reperfusion renal injury

**DOI:** 10.1186/s40709-020-00125-2

**Published:** 2020-09-29

**Authors:** Maria Tsivilika, Eleni Doumaki, George Stavrou, Antonia Sioga, Vasilis Grosomanidis, Soultana Meditskou, Athanasios Maranginos, Despina Tsivilika, Dimitrios Stafylarakis, Katerina Kotzampassi, Theodora Papamitsou

**Affiliations:** 1 Faculty of Medicine, School of Health Sciences, Aristotle University of Thessaloniki, Gianni Chalkidi 45, Charilaou, 54249 Thessaloniki, Greece; 2 1st Department of Internal Medicine, Faculty of Medicine, AHEPA University Hospital, Aristotle University of Thessaloniki, Thessaloniki, Greece; 3 Department of Surgery, AHEPA University Hospital, Aristotle University of Thessaloniki, Thessaloniki, Greece; 4 Department of Colorectal Surgery, Addenbrooke’s Hospital, Cambridge, UK; 5 Laboratory of Histology- Embryology, Faculty of Medicine, School of Health Sciences, Aristotle University of Thessaloniki, Thessaloniki, Greece; 6 Independent Researcher, Thessaloniki, Greece; 7 Independent Researcher, Thessaloniki, Greece; 8 2nd Department of Urology of Aristotle University of Thessaloniki, Papageorgiou General Hospital, Thessaloniki, Greece

**Keywords:** Kidney, Ischemia reperfusion, Cardiac arrest, Renal injury, Acute tubular necrosis, Immunohistochemistry, Adaptive immune response

## Abstract

**Background:**

The present study aims to investigate, immunohistochemically, the role of the adaptive immune response in cardiac arrest/resuscitation-induced ischemia–reperfusion renal injury (IRI), namely to assess the presence of lymphocytes in renal tissue samples and the connection between the extent of the damage and the concentration of the lymphocytes by comparing the kidneys of non resuscitated swine with the kidneys of resuscitated swine.

**Methods:**

Twenty four swine underwent cardiac arrest (CA) via a pacemaker wire. After 7 min, without any intervention, Cardiopulmonary Resuscitation, CPR, was commenced. Five min after CPR was commenced advanced life-support, ALS. Animals were divided into resuscitated animals and non resuscitated animals. Tissue samples obtained from the two groups for immunohistological study aiming to detect T-cells, B-cells and plasma cells using CD3 + , CD20 + , and CD138 + antibodies.

**Results:**

There seems to be a strong concentration of T lymphocytes in the kidney tissues after ischemia of both non-resuscitated and resuscitated swine. B lymphocytes, also, appear to have infiltrated the ischemic kidneys of both animal groups; nevertheless, the contribution of T lymphocytes to the induction of injury remains greater. There is no strong evidence of correlation between the plasma cells and the damage.

**Conclusion:**

The adaptive immune response seems to have a strong association with kidney injury and acute tubular necrosis after cardiac arrest/ resuscitation-induced ischemia–reperfusion. However, the extent to which the adaptive immune cells are involved in the induction of renal injury remains uncertain and there are many questions about the mechanism of function of these cells, the answers of which require further studies.

## Background

Acute tubular necrosis (ATN) and subsequent renal failure induced by ischemia–reperfusion injury (IRI) or sepsis remain the leading cause of morbidity and mortality among patients in the intensive care unit [1]. ATN after ischemic shock has a mortality rate of 30% and many survivors are subject to dialysis [2]. Among patients surviving cardiac arrest (CA), the onset of acute kidney injury (AKI) is also common (> 75%) and persistent AKI (PAKI) occurs in more than one-third of the patients [3]. A plethora of renal diseases, but also renal transplant rejection [4] are due to AKI resulting from ischemic shock [5]. Both the innate immune response and the adaptive immune response could exacerbate renal tissue damages and create permanent damage [6]. In hypoxia, endothelial and tubular epithelial renal cells do not absorb the oxygen that is necessary for their survival. Due to the lack of oxygenation, it is impossible to remove and metabolize the cellular waste, the accumulation of which leads to apoptosis tubular cells and finally to ATN [7]. Upon reperfusion, blood components detect the injured renal tubular cells and their signaling factors and trigger the inflammatory response [8–11]. Extended renal inflammation can lead to renal tissue fibrosis and acute renal injury [12–16].

Leukocyte cells are the most associated cells with renal IRI and most of the studies focus on their activity and their involvement in kidney inflammation. Leukocytes are involved in causing kidney damage through their phagocytic activity, the release of chemotactic cytokines related to ischemic damages, and the release of reactive oxygen species. The above contributes to the induction of renal microangiopathy and ultimately to the occurrence of renal IRI [8, 17]. Many substances that influence leukocyte influx or activation, such as neutrophil elastase [18], the tissue-type plasminogen activator [19], activated protein C [20, 21], hepatocyte growth factor [22, 23], and CD44 [24], appear to be involved in the induction of renal injury. Although the cells of innate immunity are the cells that are directly associated with kidney inflammation and damages, however, the present study provides strong evidence that the adaptive immunity cells are, also, directly involved in CA/resuscitation-induced renal IRI.

The role of adaptive immunity in non-infectious kidney injuries such as ischemic kidney injury is a surprise concerning the lymphocyte activation and function since the alloantigen is absent. The hypoxia/re-oxygenation process may be sufficient for lymphocyte activation, [25]. Distress signals produced by injured tissue following ischemia appear to trigger an innate immune response mechanism that recruits all available immune modes to respond to injury [26]. Expression of the chemokine RANTES (Regulated upon Activation, Normal T Cell Expressed and Presumably Secreted) is likely to be a mechanism of T cell activation in the absence of an alloantigen and its regulation has been associated with the induction of renal injury by T cells [28]. The laparotomy procedures, also, accelerate systemic inflammatory response and therefore the early infiltration of T and B lymphocytes into kidneys.

T lymphocytes are found in post-ischemic kidney biopsies and appear to be a key mediator in the induction of renal IRI [29]. The involvement of T lymphocytes in renal IRI predisposes to the possible involvement of B lymphocytes as well. There is evidence that B lymphocytes are involved in damage after ischemia–reperfusion through the production of pathogenic IgM [30]. Studies on infectious diseases show a strong association of T cell function with B lymphocytes [31, 32]. B lymphocytes deficient mice exhibit abnormalities in their organogenesis and organic function throughout their development [33]. T lymphocytes, as well as B lymphocytes, intervene in various stages of renal failure by ameliorating damage following ischemia. Experimental studies in knockout mice have shown that the deficiency of either T or B lymphocytes has a protective effect against renal injury [34–36]. Of particular note were the findings of an experimental study in RAG-1 (Recombination activating gene 1) knockout mice. These mice lack both T and B lymphocytes but nonetheless showed no resistance to renal injury induced by ischemia, and the transfusion of T and B lymphocytes to these mice resulted in significant kidney protection. It is therefore concluded that while B or T cell deficiency alone leads to a decrease in IRI, the combined deficiency of both is not protective [36]. In addition, the transfer of activated lymphocytes from mice to mice lacking T lymphocytes that are subjected to acute renal injury after ischemia, 24 h after reperfusion, appears to have a protective effect against IRI [37]. The above findings highlight the complex and combinatorial interactions that take place between lymphocytes which defining their actions. CD28 T cell protein, as well as its binding to the B7-1 ligand (CD80), also appears to be strongly associated with IRI by T lymphocytes [38].

The NKT cells also seem to involve in IRI. NKT cells are found in ischemic kidneys already 3 h after reperfusion [37]. Activation of NKT cells increases renal IRI by triggering neutrophil infiltration into renal tissue and production of IFN-γ interleukin by both NKT cells and neutrophil cells [39]. Except for the IFN-γ, NKT cells express, also, interleukins such as IL-4 and IL-10. Experimental studies in mice show that administration of isoflurane, which inhibits the infiltration of NKT cells, neutrophils and macrophages into the renal tubules, attenuates the acute renal IRI [40].

In the present study (conducted in resuscitated and non-resuscitated swine), it is intended to investigate, immunohistochemically, the role of the adaptive immune response in CA/resuscitation-induced renal IRI, namely to assess the presence of T cells, B cells and plasma cells in renal tissue samples. The presence of these cells was confirmed immunohistochemically using CD3 antibody staining, for the T cell detection, CD20 antibody staining, for the B cell detection, and CD138 antibody staining, for the plasma cell detection. This study, also, aims to determine the connection between the extent of the damage and the concentration of the lymphocytes by comparing the kidneys of not resuscitated swine with the kidneys of resuscitated ones.

## Methods

### Experimental protocol

The experiments were performed at the Surgical Research Laboratory of AHEPA Hospital, Aristotle University of Thessaloniki, Greece. Twenty four female Munich swine (3 months old, 23.0 ± 2.9 kg BW) were premedicated, intubated and mechanically ventilated as previously described [41, 42]. The femoral artery and both femoral veins were cannulated and an 8Fr sheath, and, through that, a 7.5Fr Swan-Ganz CCOmboV catheter (Edwards Lifesciences, Irvine, CA, USA), was inserted in the right femoral artery of each animal for hemodynamic monitoring. All animals underwent CA via a pacemaker wire placed through the Swan-Ganz catheter. After 7 min without any intervention, cardiopulmonary resuscitation (CPR) was attempted with chest compressions using the LUCAS CCS (chest compression system) CPR device (Stryker Medical, MI, USA). Five min after CPR was commenced, advanced life-support (ALS) measures were undertaken with rate analysis, defibrillation and/or drug administration (all animals were given vasodilators inotropic drugs as it is required by the ALS protocol), as per the European Resuscitation Council (ERC) 2015 Guidelines, for 40 min. Animals were divided into two groups, according to the return of spontaneous circulation (ROSC) or not. Fourteen out of 24 pigs belong to the swine whose systemic circulation did not return after ALS (symbolized as no ROSC), while 10 out of 24 swine belong to the swine to which the systemic circulation returned after ALS (symbolized as ROSC). In non-resuscitated animals (no ROSC after 40 min), CPR was stopped and tissue samples obtained for histology. In resuscitated animals (ROSC), the animals were supported for 4 h and then sacrificed with thiopental and potassium chloride administration (KCl) and tissue samples were also obtained for optical microscopy observation. The advantage of this study is that the experiment was performed on swine that constitute large animals and their systematic circulation, as well as their immune system, simulate these of humans.

### Immunochemical staining

Kidney tissue samples were taken, using a scalpel, to 3 mm thick, 20 × 30 mm. The samples were placed in a formol solution, 10% formaldehyde, for 24 h. The histokinetic protocol process took place, the process lasted 14 h, in order that the tissue samples pass the stages of fixation, dehydration, clearing, and finally the paraffin infiltration at 60 °C that is necessary for the sample preparation. After the histokinetic procedure, the tissues were paraffin-embedded by placing them on a tissue embedding machine. Finally, tissue-paraffin blocks were microtomed into three micrometer-thickness (3 μm) tissue sections. Some sections were stained with Hematoxylin and Eosin, EH, and then adjacent sections were placed on positively charged slide plates for immunohistochemical staining via VENTANA system, (Ventana Medical Systems, Arizona, USA).

For the immunohistochemical study the VENTANA BenchMark XT computerized automated system (Ventana Medical Systems, Arizona, USA) was used with the ultra-View Universal DAB Detection Kit, (Ventana Medical Systems, Arizona, USA). The water bath was set to 60 °C as it contained 90% water and 10% ethanol (100%). The sample was incubated in the oven for 60 min at 60 °C. The ultra-View Universal DAB Detection Kit detects specific antibodies bound to an antigen in paraffin-embedded tissue sections.

The reagents and antibodies used were:

1. **CD3 + **(mouse monoclonal antibody, Ventana company, Arizona, USA) at dilution 1/100 v/v and incubation time 42 min for T-lymphocytes detection.
2. **CD20 + **(mouse monoclonal antibody at Antibody Diluent solution, of Ventana company, Arizona, USA) at dilution 1/100 v/v and incubation time 42 min, for B lymphocytes detection.
3. **CD138 + **(mouse monoclonal antibody, Ventana company, Arizona, USA) at dilution 1/50 v/v and incubation time 42 min. CD138 is an immunohistochemical marker for plasma cells.

The above mentioned immunohistochemical staining procedure was repeated twice for each of the three antibodies that were examined. All specimens were examined under optical microscope (Zeiss, Jena, Germany), by two independent researchers, and photographs were taken using a Contax camera (Yashica, Hong-Kong, China) attached to the microscope.

Immunohistochemical staining according to the intensity and extent of the stained specimen in the slide plate is characterized as shown in Table 1. The calculation of the percentage of the stained specimen took place via a microscope cross table and resulting from the average percentage of the subjective crisis of the two independent researchers who observed the samples to assess the intensity and the extent of staining.

**Table 1:** Immunostaining intensity classification

Characterization	Symbolism	Staining intensity	Stained specimen
Negative	0	No staining reaction	< 20%
Weakly positive	1	Weak staining intensity	20–50%
Moderate positive	2	Moderate staining intensity	51–80%
Strongly positive	3	Strong staining intensity	> 80%

### Hypothesis

#### Testing

One of the aims of this study is to evaluate the statistically significant difference between the non-resuscitated and the resuscitated swine, for the presence of the T-, B- and plasma cells. The presence of these cells was examined by the intensity of each of the three immunochemical antibody stainings that were used for the detection. The CD3, CD20 and CD138 antibody infiltration compared between the specimens of the non-resuscitated swine and the specimens of resuscitated swine. The two-sample t-test for unequal sample sizes with similar variances was used. The alternative hypothesis is that the average value for each antibody infiltration for non-resuscitated swine is higher than the average value for each antibody infiltration for resuscitated swine.

Thus, the null hypothesis is that the average value for each antibody infiltration for non-resuscitated swine is smaller or equal than the average value for each antibody infiltration for resuscitated swine.

$${H}_{A}: {\mu }_{1}>{\mu }_{2}$$

$${H}_{n}: {\mu }_{1}\le {\mu }_{2}$$

The t statistic to test whether the means are different can be calculated as follows:

$$t = \frac{{\overline{{X_{1} }} - \overline{{X_{2} }} }}{{s_{p} \cdot \sqrt {\tfrac{1}{{{\text{n}}_{1} }} + \tfrac{1}{{{\text{n}}_{2} }}} }}$$

where

$${s}_{p}=\sqrt{\frac{\left({n}_{1}-1\right){s}_{{X}_{1}}^{2}+({n}_{2}-1){s}_{{X}_{2}}^{2}}{{n}_{1}+{n}_{2}-2}}$$

The swine whose systematic circulation has not returned and which number 14 are represented by sample 1, while the swine whose systematic circulation has returned and number 10 are represented by sample 2 (Table 2).

**Table 2:** Statistical data

	n_1_	n_2_	X_1_	X_2_	s_1_	s_2_
CD3	14	10	0.79	0.68	0.03	0.11
CD20			0.28	0.16	0.09	0.10
CD138			0.05	0.02	0.06	0.03

## Results

**Fig. 1:**
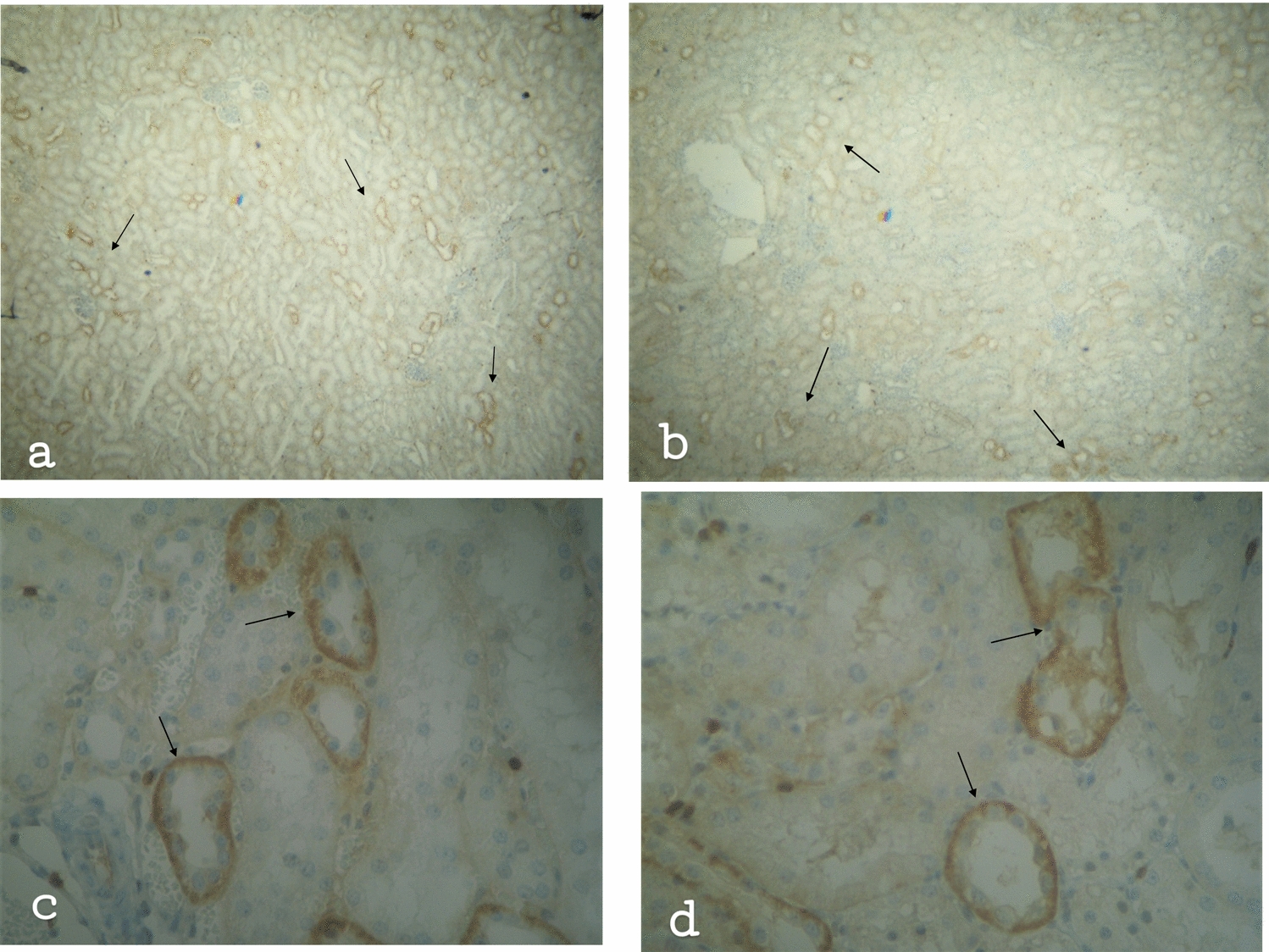
**a** Kidney of non-resuscitated swine. T lymphocytes positive tubular epithelium (↑). Few T lymphocytes in connective tissue, × 16. **b** Kidney of resuscitated swine. T-cell positive epithelium (↑). Few T lymphocytes in connective tissue, × 16. **c** Kidney tissue of non-resuscitated swine. Intensively positive tubules (↑). T lymphocytes among epithelial cells with a main concentration above the basement membrane, × 160. **d** Kidney of resuscitated swine. Damaged tubules strongly positive to T-cells (↑), × 160

**Fig. 2:**
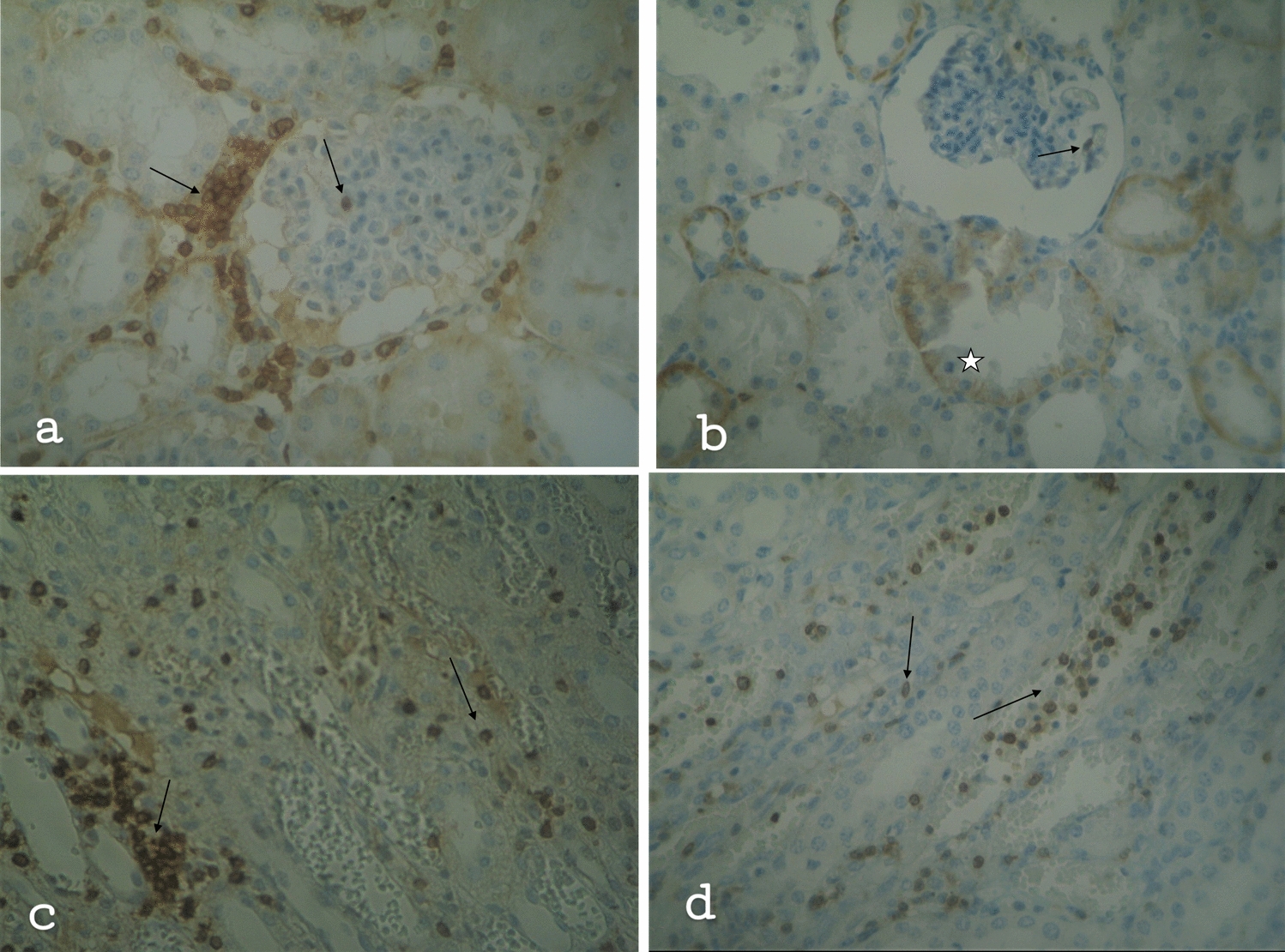
**a** Kidney cortex of non-resuscitated swine. Intense accumulation of T lymphocytes around the renal corpuscle (↑) T cell positive Bowman’s capsule and glomerulus, × 160. **b** Kidney cortex of resuscitated swine. Few T lymphocytes within the glomerulus of the renal corpuscle (↑). Beneath the renal corpuscle, damaged proximal tubule stained at the basement membrane (☆). Fewer T-cells than the non-resuscitated swine, × 160. **c** Kidney medulla of non-resuscitated swine. T lymphocytes around the tubules (↑) and in the connective tissue (↑), × 160. **d** Kidney medulla of resuscitated swine. Plenty of T cells into the damaged tubule (↑). Few T cells in the connective tissue (↑), × 160

In brief, Figs. 1a–d and 2a–d illustrate the following: the T- cells concentration observed into the connective tissue, and around the renal tubules, especially the proximal convoluted renal tubules. T lymphocytes are located mainly above the basement membrane of the tubular epithelium as well as among the renal tubular epithelial cells. CD3 + T-cells were also detected around the renal corpuscles but also within the glomerular capillaries and the Βowman capsule. CD3 + T lymphocytes identified not only in the cortex but inside the kidney medulla as well. In resuscitated swine, the staining is milder and the morphological damages are less.

**Fig. 3:**
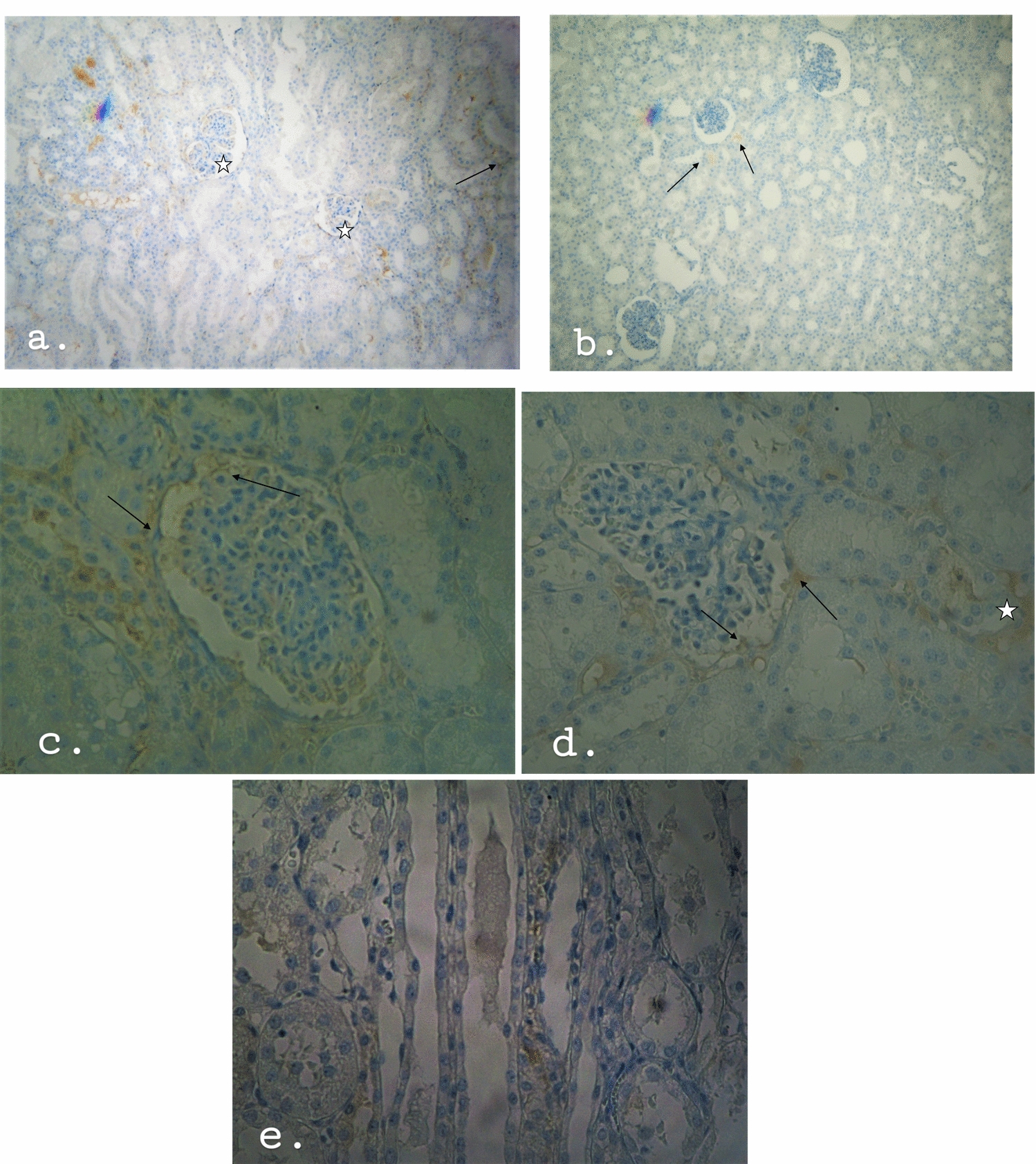
**a** Kidney of non-resuscitated swine. Few B cells around the tubules (↑) and mild staining to negative renal corpuscles (☆), × 40. **b** Kidney of resuscitated swine. Few, almost none, B-cells in connective tissue (↑), × 40. **c** Kidney cortex of non-resuscitated swine. Few B-cells in the parietal layer of the Bowman’s capsule of the renal corpuscle (↑)and in the urinary space (↑), × 160. **d** Kidney cortex of resuscitated swine. Few B-cells in the parietal layer of the Bowman’s capsule of the renal corpuscle (↑) and within the connective tissue (☆), × 160. **e** Negative kidney medulla of non-resuscitated swine, × 160

It is found that B-cells are mainly located in the parietal layer of the Bowman’s capsule of the renal corpuscles and in the urinary space (Fig. 3a–e). B-cells are also found among the renal tubular epithelial cells, mainly the proximal tubules, as well as into the connective tissue. The B-cells volume and concentration are much less than the T cells. In contrast to T-cells, no B-cells were found in the kidney medulla. In resuscitated swine, the staining is weakly positive, even negative and the concentration of B cells is lower than in non-resuscitated swine. In addition, morphological damage is less.

**Fig. 4:**
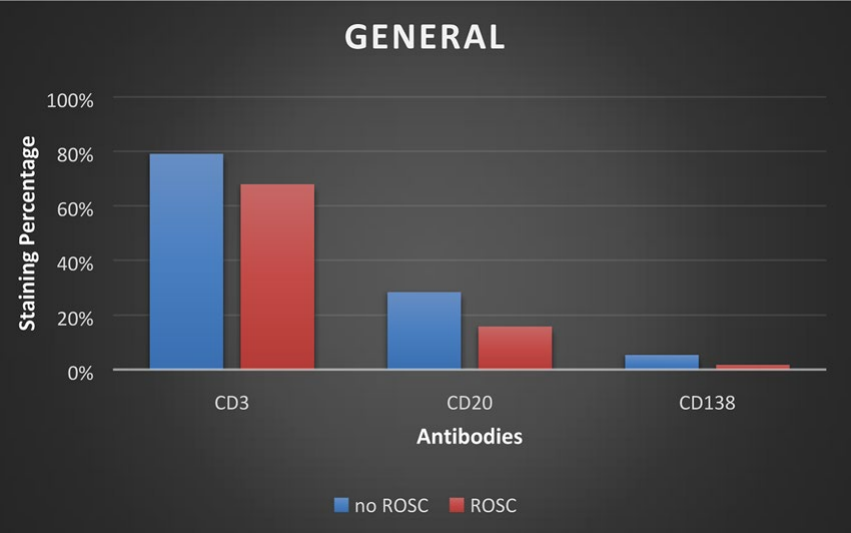
The T-cell, B-cell and plasma cell infiltration as determined by immunostaining of post‐ischemic kidney tissue with CD3, CD20 and CD138 antibodies (mouse monoclonal antibody, Ventana company, Arizona, USA). Ten resuscitated and 14 non-resuscitated stained specimens were viewed and counted for positively stained cells. Infiltration at non-resuscitated specimens (blue bars) at 24 h post‐ischemia was increased compared to the infiltration at resuscitated specimens (red bars). Both in non-resuscitated and resuscitated specimens, the infiltration of T-cell was greater than the infiltration of B-cell while there is no strong evidence of plasma cell infiltration

The concentration and staining volume of plasma cells is less, to negative compared to the the concentration and staining volume of T and B lymphocytes (Fig. 4a–d). The non-resuscitated swine showed mild to negative staining, (staining 0), with few plasma cells dispersed in the connective tissue, the parietal layer of the Bowman’s capsule of the renal corpuscles and in the urinary space, as well as in and around the capillary vessels. Resuscitated swine was negative to plasma cells, (staining 0).

### Statistical results

Statistical analyses showed that the t-value is higher than the critical value for CD3 (*p* = 0.0065) and CD20 (*p* = 0.0017) antibodies, and therefore, the null hypothesis is rejected. Consequently, the difference between the two samples is statistically significant, for T-cells and B-cells, enhancing the association of cardiac arrest/resuscitation-induced ischemia–reperfusion renal injury with the T- and B-cell infiltration, into the kidney tissue, assuming that the non-resuscitated swine developed greater damages. For CD138 antibody, the t-value is less than the critical value, failing to reject the null hypothesis (*p* = 0.0536). Therefore, the difference between the 2 samples, for plasma cells, is not statistically significant, reinforcing the immunohistological observations which also supporting that there is no strong evidence of involvement of the plasma cells in CA/resuscitation-induced renal IRI (Table 3).

**Table 3:** Statistical results

	Sp	t-statistic	df	C_0.05_	C_0.025_
CD3	0.07	3.69	22	1.717	2.07
CD20	0.09	3.29			
CD138	0.05	1.68			

## Discussion

**Fig. 5:**
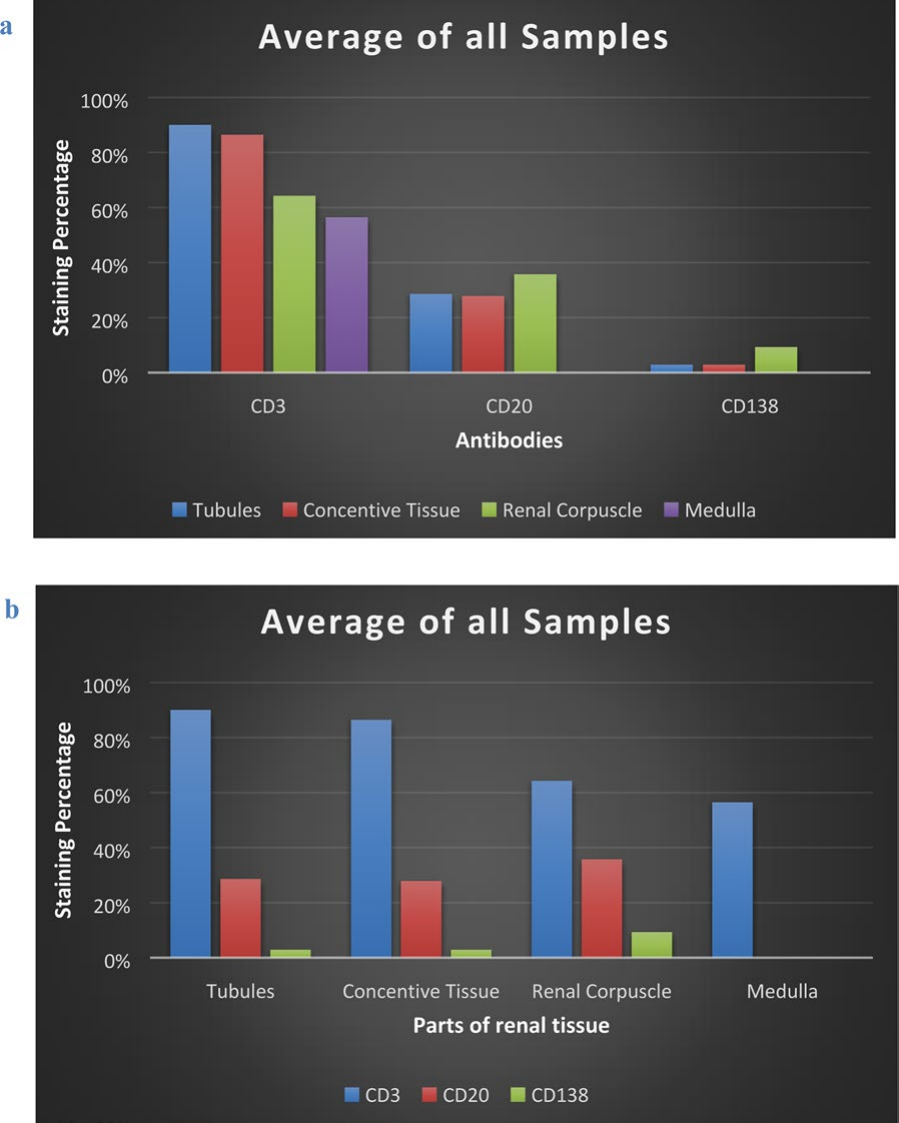
**a**, **b** Antibody CD3 staining showed that T cell infiltration is strongly positive throughout the kidney tissue (staining 3), except for the renal corpuscle and the medulla where it is moderately positive (staining 2). CD20 antibody staining (to wit, B cell infiltration) is weakly positive. The weak concentration appears mainly around the renal corpuscle (staining 1) while the medulla is negative to CD20 antibody (staining 0). CD138 antibody staining, therefore the plasma cell infiltration is negative (staining 0)

In resuscitated swine, the concentration of T-cells is lower than the concentration in non-resuscitated swine (Fig. 4). In addition, morphological damage is not that evident at resuscitated swine (when compared with non-resuscitated group). It is concluded that swine which recovered, and also appear with less damage, also exhibits less T-cell infiltration, further reinforcing the association of T-cells with AKI following ischemia. Ischemic mice and rats treated with drugs that prevent T-cell infiltration into the kidneys have decreased renal impairment [43, 44]. Mice lacking T lymphocytes are protected from IRI while an increase in renal IRI has been observed following the administration of T lymphocytes to these animals [45]. The observation that the main staining of cells occurs around the renal tubules (Fig. 5) and especially the proximal convoluted tubules of the kidney, enhances the aspect that the proximal tubular cells are the most vulnerable to renal ischemia [46]. Renal tubules bearing damaged epithelium and damaged renal corpuscle show higher CD3 + T lymphocyte concentration, which underscores the involvement of T lymphocytes in damage exacerbation. Studies in CD4 + /CD8 + knockout mice had significantly impaired renal IRI compared to wild type mice [29]. Experimental studies in only CD4 + knockout mice also limit kidney damage following ischemia–reperfusion, emphasizing that CD4 + T lymphocytes, and in particular CD4 + Th1 T lymphocytes, which produce IFN-γ, is the major subset of T-cells that involve in renal IRI [45]. This claim is supported by the fact that STAT4 (Signal transducer and activator of transcription 4) deficient mice showed improvement of renal IRI compared to STAT6 (Signal transducer and activator of transcription 6) deficient mice [47]. The detection of T lymphocytes in the glomerular capillaries and the Bowman’s capsule suggest that the action of T cells is not restricted to signal transduction via the cytokine exudation but rather by the infiltration into the kidneys and the local action. CD11/CD18 as well as the intercellular adhesion molecule 1 (ICAM-1) have been studied for their role in adhesion of leukocytes to endothelium and ultimately their involvement in the induction of renal injury, mainly by neutrophil adhesion [48, 49]. However, these above adhesion pathways also mediate lymphocyte adhesion [50]. Phorbol ester treatment in vivo in mouse renal system increased the adhesion of T lymphocytes to renal tubular epithelial cells (RTEC). T-cell adhesion to RTECs increased even further after exposure of the system to hypoxia-reoxygenation conditions, a technique that mimics ischemia–reperfusion conditions [29]. The detection of T lymphocytes, also, inside the kidney medulla underlined the in-depth adhesion of T lymphocytes.

Although, B-cells are involved in the induction of IRI, the contribution of T lymphocytes to the induction of injury remains greater (Fig. 5). The location of B-cells, in the parietal layer of the Bowman's capsule of the renal corpuscle and in the urinary space, suggests that the function of the B-cells are not confined to the transduction of signals to the kidneys but rather the B lymphocytes infiltrate the kidneys and act locally, such as the T-cells. The fact that kidney medulla was positive only for T lymphocytes underscores the lower involvement of B-cells in renal IRI compared to T-cells. Resuscitated swine seems to have fewer B-lymphocytes and less damage than the non-resuscitated swine (Fig. 4). These data lead to the conclusion that swine whom the systemic circulation returned, which also appear to have fewer damages, also exhibit less B-cell infiltration. The above seems to strengthen the association of B-cells with AKI that follows ischemia. It is uncertain whether the plasma cells are involved in the renal IRI, such as B and T lymphocytes. B lymphocytes deficient mice showed better renal function and less tubular damage after ischemia–reperfusion compared at 24, 48 and 72 h after ischemia, compared to wild type mice. Leukocyte and T lymphocyte infiltration in the 2 groups of mice did not differ significantly. Serum administration of antibodies to knockout mice had a therapeutic effect against the injury whereas B-cell administration showed no improvement [35]. It is concluded that plasma cell serum may be involved as an auxiliary defense mechanism to limit the damage when the damage is large, as in not resuscitated swine. Consequently, there is no strong evidence which correlates the plasma cells with the damage (Fig. 5).

Hopefully, the experimental results of this study could be used for better identification of the kidney damage in patients with renal IRI, such as patients with myocardial infarction, patients after cardiopulmonary bypass, and "bypass" aortic surgery, patients with sepsis, or renal trauma. The present study aims to be a useful tool for predicting renal allograft functional capacity, as well as for more precise determination of the time a kidney may remain out of the systemic circulation in selective urological interventions such as partial renal resection and tumor removal in cases of neoplasia. Serum creatinine is a reliable indicator of renal glomerular filtration (GFR) and is used for checking the renal function [51].

## Conclusion

The findings of the immunohistochemical examination suggest a strong concentration of T lymphocytes in the kidney tissues after CA/resuscitation-induced IRI of both non-resuscitated and resuscitated swine. The study emphasizes that the accumulation of T-cells is proportional to the damage observed in the tissue. B lymphocytes, also, appear to infiltrate the ischemic kidneys of both non-resuscitated and resuscitated swine while there is no strong evidence which correlates the plasma cells with the damage. However, the extent to which the adaptive immune cells are involved in the induction of renal injury remains uncertain and there are many questions about the mechanism of function of these cells, the answers of which require further studies.

## Data Availability

The datasets used and/or analysed during the current study are available from the corresponding author on reasonable request.
